# Study of the Prevalence of Obesity and Its Association with Maternal and Neonatal Characteristics and Morbidity Profile in a Population of Moroccan Pregnant Women

**DOI:** 10.1155/2021/6188847

**Published:** 2021-12-14

**Authors:** Fatima Taoudi, Fatima Zahra Laamiri, Fatima Barich, Nadia Hasswane, Hassan Aguenaou, Amina Barkat

**Affiliations:** ^1^Mother and Child Couple Health and Nutrition Research Team, FMP de Rabat, Mohammed V University, Rabat, Morocco; ^2^Hassan First University, Higher Institute of Health Sciences of Settat, Health Sciences and Technology Laboratory, Settat, Morocco; ^3^Joint Unit of Nutrition and Food Research, CNESTEN–Ibn Tofaιl University–URAC 39, Regional Designated Center for Nutrition (AFRA/IAEA), Rabat, Morocco; ^4^Higher Institutes of Nursing Professions and Health Techniques, Rabat, Morocco

## Abstract

Obesity is a real public health problem whose prevalence continues to increase throughout the world. It affects all age groups and does not spare pregnant women. This work aims to determine the prevalence of obesity and to study its association with maternal and neonatal characteristics and the morbidity profile of pregnancy. This is a descriptive and cross-sectional study carried out in the maternity ward of the prefectural hospital center called “Sidi Lahcen” in Témara, Morocco, over a 12-month period. Maternal and neonatal data are collected through a preestablished questionnaire, and anthropometric parameters were recorded. 390 participants, aged between 18 and 43 years, were included in this study, with a prevalence of overweight and obesity of 34.9% and 41%, respectively. Correlation results revealed that the prevalence of overweight and obesity was significantly elevated in women over 25 years (*p* < 0.001). The rate of caesarean section was four times higher in obese women compared to women of normal weight (53.8% versus 12.8%; *p*=0.018). The over-term was significantly high in the obese group compared to the nonobese group (33.8% versus 20.2%; *p*=0.013). A statistically significant positive correlation was found between gestational body mass index and newborn birth weight (*r* = 0.29; *p* < 0.001) as well as a high prevalence of macrosomia in newborns of comparatively obese women compared to newborns of nonobese women (17.6% versus 9.6%; *p*=0.041). The correlation analysis with the morbidity profile showed a significantly high preponderance of gestational diabetes, anemia, and toxemia of pregnancy in the obese group compared to the normal group (*p* < 0.001). This study clearly demonstrated that obesity during pregnancy is associated with higher risks of maternal and neonatal complications, the management of which places a burden on the health system as well as families. These data reinforce the need to improve antenatal care for the prevention of obesity and its preventable complications.

## 1. Introduction

Obesity has been considered by the WHO as a global epidemic since 1998. It is a real public health problem. Its global prevalence has almost tripled over the past four decades and continues to rise in developed and developing countries [1, 2].

In 2016, more than 1.9 billion adults (18 years and older) were overweight. Of this total, more than 650 million were obese [3]. Today globally, overweight and obesity are linked to more deaths than underweight.

There are more obese than underweight people in all regions except parts of sub-Saharan Africa and Asia [3]. Obesity leads to serious health consequences, in particular by the increase in cardiovascular diseases, type 2 diabetes, musculoarticular disorders, and a few cancers (breast, endometrium, and colon), but also through moral harm as a result of the discrimination it entails [4, 5].

Since obesity does not spare pregnant women, it is an obstetric risk as well as antenatal factor prepartum or postpartum, both on the fetal and the maternal side [6, 7].

It is responsible for a large number of maternal and fetal complications. Obese patients have three times more infertility problems than normal weight women [7], in addition to a significant increase in the rate of abortions before 20 weeks of amenorrhea (SA), regardless of the mode of conception in patients [8].

In addition, overweight and obese women are prone to gestational diabetes, preeclampsia, and genital and urinary tract infections [9]. During work, obesity is associated with lengthening the duration of the first stage and a significant decrease in uterine contractility [10, 11].

As for postpartum complications, overweight or obese women are at risk of hemorrhage from delivery and massive postpartum hemorrhage [9]. Furthermore, weight gain during pregnancy accounts for 20–35% of long-term changes in maternal weight [12].

This cannot be a burden on the health system associated with higher levels of postpartum maternal weight retention and an unwanted cardiometabolic risk profile in the offspring [13, 14].

For fetal complications, newborns with obese mothers more often suffer from neonatal distress, meconium inhalation, and neonatal death [15]. They are about twice as likely to have fetal macrosomia [9, 16].

In Morocco, the prevalence of obesity is high among women. Indeed, obesity was detected in 20% (18.9–21.1) of respondents, three times more common in women 29% (27.4–30.6) compared to men 11.0% (9.5–12.6) (MS, 2018) [17].

In addition, numerous studies have shown a strong influence of socioeconomic status on obesity, especially in women, causing changes in their behaviors that alter their energy intake and energy expenditure and therefore affect their body fat storage [18, 19].

However, studies of obesity during pregnancy are few, while obesity in pregnant women poses many obstetric and perinatal problems.

In this context, our present work proposes to determine the prevalence of obesity and to study its association with maternal and neonatal characteristics and the morbidity profile of pregnancy.

## 2. Materials and Methods

### 2.1. Type of Study

This is a cross-sectional descriptive study that was carried out at the maternity ward of the prefectural hospital center “Sidi Lahcen” in Témara, Morocco, over a 12-month period from October 2018 to September 2019.

### 2.2. Study Population

The study concerned a population of pregnant women who gave birth in this maternity ward.

### 2.3. Inclusion and Exclusion Criteria

We included in this study all parturients with a physiological pregnancy who gave birth in this hospital during the study period and who agreed to participate in our survey.

The study excludes women who do not know their weight before pregnancy. Similarly, women who had a serious complication of childbirth, women with a mental pathology, women who do not consent, and women with whom the pretest was performed were excluded from the study.

### 2.4. Sampling Method

The sampling method used in this study is of the probability type. The survey was carried out on all the women who gave birth in the maternity ward of the Sidi Lahcen regional prefectural hospital in Témara of the prefectural hospital center “Sidi Lahcen” in Témara. Based on the Lorenz formula developed by Cochran and Ardilly [20, 21], *n* = *t*2 × *p* × (1 – *p*)/m^2^, where *t* = 1.96, and based on a regional prevalence of 14.4% [22] and an accuracy of 5%, a minimum population of 189 are required to obtain statistically representative data. In this work, we recruited a global sample of 390 pregnant women.

### 2.5. Data Collection Methods

Data collection is carried out by a face-to-face interview with the women via a preestablished questionnaire tested and validated by experts in perinatal care (research team in health and nutrition of the mother and child couple, Faculty of Medicine and Pharmacy of Rabat, Mohammed V Rabat University).

The investigation explored several aspects, including the following:

(a) Demographic and socioeconomic data (age of mothers, marital status, place of residence, level of education, occupation of mother and head of household, monthly income, etc.).
(b) Medical and obstetric history (chronic pathology, history of diabetes, number of pregnancies, number of abortions, neonatal death). In addition, the study was supplemented by means of the obstetrical file which made it possible (blood glucose, complete blood count (CBC)), monitoring of the pregnancy, the number of prenatal consultations, and gestational age.
(c) Maternal anthropometric data.
Women in the study were chosen based on knowledge of their pregestational weight. Then the anthropometric data were collected on admission, namely,
    Weight: the weight of the women is taken with a portable scale, scale of the type SECA, maximum load 150 kg, and minimum load 10 kg. The reading is made to within 0.5 kg.
    The women are weighed with the least amount of clothing possible, in a standing position, well straight, the arms in the extension of the body. The scale is calibrated regularly and is calibrated several times during each session.
    Waist: it has been taken with the help of a graduated height up to 2 m. The women are measured barefoot, the heels joined, the arms extended along the body, the heels, the shoulders, and the buttocks touching the height. The lower edge of the orbits of their eyes in the plane of Frankfurt.
    The reading is done to within 0.1 cm. Women are asked to remove their scarf before performing this measurement.
    Calculation of BMI: the gestational body mass index of parturients was calculated by dividing weight over height squared. Then, the women were further subdivided according to WHO recommendations into four BMI groups: (1) the underweight group (BMI < 18.5 kg/m^2^), (2) the normal group (18.5 ≤ BMI < 25), (3) the overweight group (25 ≤ IMC < 30), and (4) the obese group (BMI ≥ 30 kg/m^2^) [20].
(d). Neonatal data are as follows:
    Anthropometric measurements in newborns:
     Weight: the measurement is made using a calibrated flail scale with a maximum capacity of 20 kg and a minimum load of 500 g. The scale is calibrated before each weighing. The weight of newborns is taken from birth, after the first care following the birth. The child is naked, lying on the scale carrier, and its weight is determined when the scale is in balance.
    Apgar score:
     The Apgar score is a simple method for quickly assessing the health and vital signs of a newborn baby. It teaches us about the adaptation of the newborn to ectopic life. It was designed by Dr. Virginia Apgar in 1952 [23], with five criteria, namely, appearance (skin coloring), pulse (heart activity), grimace (reactivity to stimuli), activity (muscle tone), and breathing (frequency and respiratory efforts).

The Apgar score must be established in the first, fifth, and tenth minutes. When you have a score ≥7, this is a normal newborn.

An Apgar score of less than seven should lead to appropriate management, because it doubles the risk of premature death [24].

### 2.6. Ethical Consideration

The study protocol was approved by the Ethics Board of the Faculty of Medicine and Pharmacy, Mohammed V University in Rabat, Morocco (Ethical Approval number 69 delivered on 31 January 2017). Before data collection, invited participants were informed about the study objectives and methods, and both oral and written consent were obtained from all who were recruited.

### 2.7. Statistical Analysis

The statistical analysis was conducted using the SPSS epidemiological software (SPSS version 13.0). The Kolmogorov Smirnov test was used to study the normality of the distribution of quantitative variables. Thus, the quantitative variables with symmetrical distribution (age of mothers, gestational body mass index, and gestational age) were expressed as mean and standard deviation. Quantitative variables with asymmetric distribution (birth weight of the newborn) are expressed as median and quartile. Qualitative variables are expressed in numbers and corresponding percentages. The study of the association between corpulence and maternal and neonatal parameters was carried out by the Pearson khi2 test or the exact Fisher test. Pearson's correlation was used to study the association between gestational body mass index and birth weight. A threshold of *p* < 0.05 was considered significant for all analyses performed.

## 3. Results

### 3.1. Sociodemographic Profile of Women Surveyed

In this study, 390 parturients were surveyed when they were admitted in the maternity ward of the Sidi Lahcen prefectural hospital in Témara, Morocco. Analysis in Table 1 showed that the age of women varied from 18 to 43 years with an average of 29.69 ± 5.8 years and a preponderance of the 25 to 34 age group (47.4%).

The distribution by place of residence was statistically similar. In contrast, the distribution by level of education was dominated by illiterate women with a proportion of 48.5%. The distribution of women according to their spouse's income, the occupational situation, and medical coverage shows on the one hand that 57.7% had low income and on the other hand that the majority of women were inactive (91.8%) and more than half (59.5%) were without medical coverage. In addition, the majority use oral contraception (67.4%).

### 3.2. Anthropometric Data and Medical and Obstetric History of Pregnant Women

Analysis of anthropometric parameters (Table 2) showed that the average BMI of our population was 28.44 ± 4.79 kg/m^2^, with a preponderance of overweight and obesity prevalent at 34.9% and 41%, respectively. Analysis of the results in relation to the obstetric history of the women showed that the majority of women were multiparous and had no history of abortion (75.4%) and fetal death (93.1%). Of those with a medical history, 22.3% had a family history of diabetes, 3.6% had chronic conditions such as high blood pressure, and 3.6% had chronic conditions such as high blood pressure, epilepsy, and thyroid problems.

Moreover, the majority of women (97.2%) had a follow-up pregnancy either in the public sector (57%), in the private sector (21.1%), or in both (21.9%). In addition, 69.5% of women had at least 4 prenatal consultations, and 28.5% of women received iron supplementation during pregnancy.

### 3.3. Maternal and Neonatal Data

Analysis of maternal data (Table 3) revealed that the average gestational age of pregnant women in amenorrhea weeks was 39.53 ± 1.56. Cesarean delivery affected 20% of our parturients. Regarding the neonatal data, we found that macrosomia affected 15.6% of newborns and that the Apgar score at 5 min was less than 7 in the majority of newborns (71.5%).

### 3.4. Study of the Association between Body Size and Maternal and Neonatal Parameters

Table 4 showed the correlation between body size and maternal and neonatal parameters.

Analysis of the results revealed that the overall prevalence of obesity was significantly higher (*p* < 0.001) among women aged over 35 years (53.6%) compared to women aged 25 to 35 years (45.4%) and women under 25 (22.2%).

On the other hand, no statistically significant differences were observed among female respondents (mother's work, educational attainment, and area of residence).

Regarding obstetric parameters, the prevalence of obesity was significantly high in multiparous women compared to first-time women (51% versus 30%; *p*=0.026).

In addition, the rate of caesarean sections was four times higher in obese women compared to normal weight women (53.8% versus 12.8%) and statistically significant (*p*=0.018). Similarly, the prevalence of obesity and overweight was significantly high among women with multiple abortions (*p*=0.041).

Furthermore, the prevalence of women who exceeded the term was significantly high in the obese group compared to the nonobese group (33.8% versus 20.2%; *p*=0.013).

In relation to the association between BMI and neonatal data, our results revealed, on the one hand, a statistically significant positive correlation between gestational body mass index and birth weight (*r* = 0.29; *p* < 0.001) and, on the other hand, a prevalence of macrosomia in obese women compared to nonobese women (17.6% versus 9.6%; *p*=0.041).

### 3.5. Pregnancy Morbidity Profile and Its Association with Body Size of the Parturients

**Figure 1:**
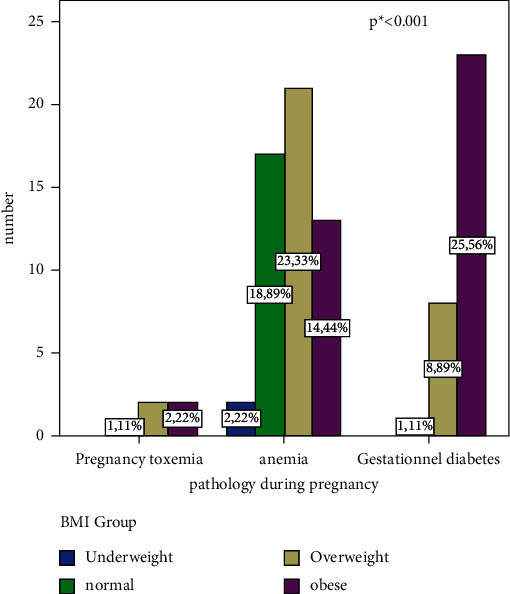
Distribution of parturients by body mass index and morbidity profile. Note: underweight: BMI < 18.5 kg/m^2^; normal: 18.5 ≤ BMI < 25 kg/m^2^; overweight: 25 ≤ BMI < 30 kg/m^2^; obese: BMI ≥ 30 kg/m^2^. ^*∗*^Fisher's exact test. A value of *p* < 0.05 is considered significant.

Our study also explored the pathologies that occurred during pregnancy. Overall analysis of the results (Figure 1) showed that 90/390 (23.1%) had at least one morbidity whose profile was dominated by anemia (58.89%) followed by gestational diabetes (35.56%) and finally gravid toxemia (5.56%). Analysis of the morbidity profile by body size showed that the percentages of women with gestational diabetes, anemia, and gravid toxemia were significantly elevated in the overweight and obese group compared to the normal group. This difference was statistically significant (*p* < 0.001).

## 4. Discussion

The present work proposes to determine the prevalence of obesity and to study its association with maternal and neonatal characteristics as well as the morbidity profile of pregnancy in the region of Témara, Morocco, an area with a high prevalence of obesity [22].

The results showed that overweight and obesity are a problem in the study population with respective prevalence of 34.9% and 41%. These results are controversial with those reported in France and Canada with respective prevalence of 23.5%, 7%, and 23%, 36% [25, 26]. Gestational weight gain remains an independent risk factor for the health of the mother and her child in the short and long term [27]. Indeed, it is associated with increased rates of gestational hypertension, gestational diabetes, caesarean delivery, and macrosomia [6].

It is accompanied by an increase in maternal adipocytic stores, often contributing to the maintenance of postpartum overweight and increasing the risk of subsequent obesity and type 2 diabetes [12, 28]. Similarly, excessive weight gain influences the development of obesity in children in the short and long term [28, 29]. The study of the correlation between body shape and maternal parameters showed that the prevalence of overweight and obesity is higher in women over 25 years of age with a statistically significant difference (*p* < 0.001, Table 4). These results are similar to those reported in a study of Swedish Ghanaian and Nigerian women [30–32]. Similarly, these findings corroborate with the findings of Garabedian et al. which reported that obesity is affecting more and more young women [33]. This explains its increasing prevalence among pregnant women [34]. In fact, this can also be explained by the accumulation of fat during pregnancy [35].

A correlation was also observed between corpulence and the education level of the participants. Indeed, 45.6% of illiterate women were obese and 32.6% were overweight (Table 4), while those with higher or secondary education had a lower rate of obesity and overweight. Although the difference in our sample was not statistically significant, our results corroborate with the literature that considers educated women to be at lower risk of obesity because they are more aware of its risks [18, 36, 37]. As for parity, a significant correlation was observed with overweight (*p*=0.026, Table 4). This finding is similar to that found in the study by Ducarme et al. [25] which reported significant values (*p* < 0.001), with prevalence of overweight and obesity of 33.1% and 35.1%, respectively.

The impact of obesity on both gestational age and the course of delivery was also raised in our study. Indeed, the study demonstrated a significant correlation between obesity and overdue pregnancy. The impact of obesity on both gestational age and delivery patterns was also raised in our study. Indeed, the study showed a significant correlation between obesity, postterm pregnancy, and caesarean section, and these results are similar to results reported by other authors [15, 31, 38]. In the same sense, other studies have shown that the increase in caesarean sections is proportional to the size, even in the absence of maternal pathology [39, 40]. Similarly, study participants with a history of abortion were obese, which is statistically significant (*p*=0.041, Table 4). Our results remain similar to a randomized study by Lashen and Fear which reported that obese women had a 1.2 times greater risk of first trimester pregnancy loss compared to a control group of normal weight women (95% CI [1.01–1.46]), and a 3.5 times greater risk of recurrent miscarriage (95% CI [1.03–12.01]) [41]. This complication is attributed to insulin resistance traditionally encountered in obese patients as well as polycystic ovary syndrome frequently encountered in this female population. Thus, this risk is likely to increase with the degree of obesity [42].

For neonatal criteria, the Apgar score at 5 min was less than 7 in 3/4 of the newborns or (71.5%). This same result has been demonstrated in many international studies [15, 43, 44], which reported a higher incidence of newborns with Apgar less than 7–5 min in the overweight female population (Class I with 18% and Class II with 19%) compared to the rest of the population (6.9%). One would think that the frequency of fetal suffering during labor would be increased in overweight women. Similarly, a positive and statistically significant mean correlation (*r* = 0.20, *p* < 0.001) was found between obesity and newborn weight. This is consistent with several studies that have reported that obese women are about twice as likely to give birth to newborns with macrosomia [9, 16, 45]. This creates a risk of weight gain in adulthood [46].

Obesity as a morbidity factor has also been exploited in this work. Indeed, our study showed that the prevalence of pregnant toxemia and gestational diabetes was significantly higher in obese women compared to normal weight women (*p* < 0.001, Figure 1). This has been demonstrated by previous studies [47, 48]. Therefore, testing in women with risk factors early in pregnancy is recommended [49].

Gestational diabetes is diagnosed in 1–3% of pregnancies and 17% of obese women [50]. Excess adipose tissue is responsible for an overproduction of adipokines, an important part of which is involved in the inflammation phenomenon. It is these same adipokines that are responsible for the metabolic imbalances that cause the complications of obesity (insulin resistance, type 2 diabetes, atherosclerosis, and arterial hypertension) [50, 51]. Several studies have concluded that toxemia is increased in obese pregnant women [52, 53]. In this sense, our results corroborate with the comments of these authors in that the pregnant toxemia was significantly higher in the overweight and obese group compared to the normal group. Obesity is indeed strongly associated with hyperlipidemia, which, by direct or indirect mechanism, damages the endothelial cells causing vasoconstriction and platelet aggregation, contributing to the preeclampsia process [54].

Regarding anemia, our study showed that the overall prevalence was 58.89% with a statistically significant preponderance in overweight group. Our results remain less alarming than those reported in neighboring Arab Maghreb countries such as Tunisia and Mauritania with prevalence of 41% and 53.1%, respectively [55, 56]. Anemia remains a global public health problem with prevalence of 38% in pregnant women [57]. It is a global target to reduce anemia in women of reproductive age by 50% by 2025 [58]. This could be done through prenatal surveillance and other research exploring the nutritional profile of pregnant women in general and obese women in particular. This component was not explored in the present study.

## 5. Conclusion

The prevalence of overweight and obesity in our population is 34.9% and 41%, respectively. Our study has clearly demonstrated that obesity is responsible for obstetrical and neonatal complications, the management of which constitutes a burden for both the health system and the families. These data reinforce the need to improve antenatal care for the prevention of obesity and its avoidable complications.

## Figures and Tables

**Table 1:** Sociodemographic characteristics among pregnant women.

Characteristics	Pregnant women *N* = 390	95% confidence interval (CI)
Age groups (years)		
Less than 25	108(27.7)	23.1–32.1
25 to 34	185(47.4)	42.6–52.6
35 and more	97(24.9)	20.5–29.2
Area of residence		
Urban	210(53.8)	48.5–59.2
Rural	180(46.2)	40.8–51.5
Level of education		
Illiterate	189(48.5)	43.3–53.3
Primary or koranic school	72(18.5)	14.6–22.8
Primary	65(16.7)	13.1–20.6
Secondary	52(13.3)	10–16.7
Higher education	12(3.1)	1.5–4.9
Occupation of women		
House wife	358(91.8)	89.2–94.6
Employed	32(8.2)	5.4–10.8
Occupation of the household head		
Without job	36(9.2)	6.2–12.3
With job	302(77.4)	73.3–81.5
Retired	52(13.3)	10–16.7
Medical coverage		
No	232(59.5)	54.6–64.1
Yes	158(40.5)	35.9–45.4
Contraception		
No	116(29.7)	25.1–34.4
Oral contraception	263(67.4)	63.1–72.1
IUD	10(2.6)	1.3–4.4
Others	1(0.3)	0.0–0.8
Pregnancy monitoring		
No	11(2.8)	1.3–4.6
Yes	379(97.2)	95.4–98.7

Note: values are expressed as number and percentage. IUD: intrauterine device.

**Table 2:** Population distribution of female participants by anthropometric information and medical and obstetric history *α*: values are expressed as number and percentage.

Characteristics	Pregnant women *N* = 390	95% confidence interval (CI)
BMI^*α*^ tranche		
Underweight	3(0.8)	0.1–1.8
Normal	91(23.3)	19.5–27.7
Overweight	136(34.9)	30.3–39.5
Obesity	160(41)	36.2–45.9
Chronic pathology^*α*^		
No	376(96.4)	94.4–98.2
Yes	14(3.6)	1.8–5.6
History of diabetes^*α*^		
No	303(77.7)	73.6–81.8
Yes	87(22.3)	18.2–26.4
Gravidity/parity^*α*^		
1 pregnancy	90(23.1)	19–27.7
2 pregnancies	153(39.2)	34.6–44.1
3 pregnancies	147(37.7)	32.6–42.3
Previous abortion^*α*^		
Neither	294(75.4)	70.8–79.7
1 abortion	71(18.2)	14.4–22.3
2 abortions	21((5.4)	3.1–7.7
3 abortions	4(1)	0.3–2.1
Neonatal death^*α*^		
No	363(93.1)	90.1–95.4
Yes	27(6 .9)	4.6–9.5
Prenatal consultation^*α*^		
Less than 4 CP	119(30.5)	25.9–35.6
4 or more CPs	271(69.5)	64.4–74.1
Iron supplementation^*α*^		
No	279(71.5)	67.2–76.2
Yes	111(28.5)	23.8–32.8

Note: BMI: underweight: BMI < 18.5 kg/m^2^; normal: 18.5 ≤ BMI < 25 kg/m^2^; overweight: 25 ≤ BMI < 30 kg/m^2^; obesity: BMI ≥ 30 kg/m^2^. CP: prenatal consultation.

**Table 3:** Birth history and neonatal characteristics.

Characteristics	Pregnant women *N* = 390	95% confidence interval (CI)
Gestational age^*β*^	39.53 ± 1.56	39.37–39.68
Mode of delivery^*α*^		
Vaginal	312(80)	75.4–83.8
Caesarean section	78(20)	16.2–24.6
Instrumentation^*α*^		
No	279(84.0)	80.1–88.0
Episiotomy	41(12.3)	9–16
Ventouse	12(3.6)	1.5–5.7
Sex^*α*^		
Female	194(49.7)	45.1–55.1
Male	196(50.3)	44.9–54.9
Weight (g)^*ɣ*^	3400[3100–3800]	3400–3450
Weight band		
Hypotrophy	15(3.8)	2.3–5.9
Normal	314(80.5)	76.7–84.6
Macrosomia	61(15.6)	12.1–19
Apgar^*α*^ at 5 min		
Apgar < 7	279(71.5)	67.2–76.2
Apgar ≥ 7	111(28.5)	23.8–32.8
Death^*α*^		
No	379(97.2)	95.4–98.7
Yes	11(2.8)	1.3–4.6

Note: *α* values are expressed as number and percentage. *β* values are expressed as mean and standard deviation. *ɣ* values are expressed as median and quartile.

**Table 4:** Distribution of female partners by sociodemographic, obstetric, and body size parameters.

Variables	Pregnant women *N* = 390				
	Underweight *n* = 3	Normal *n* = 91	Overweight *n* = 136	Obese *n* = 160	*p* ^*∗*^
Age (year)					
<25	2(1.9)	31(28.7)	51(47.2)	24(22.2)	<0.001
25 à 35	0(0)	44(23.8)	57(30.8)	84(45.4)	
≥35	1(1.0)	16(16.5)	28(28.9)	52(53.6)	
Area of residence					
Urban	2(1)	50(23.8)	73(34.8)	85(40.5)	0.982
Rural	1(0.6)	41(22.8)	63(35)	75(41.7)	
Level of education					
Illiterate	2(0)	55(21.1)	85(32.6)	119(45.6)	0.132
Primary or koranic school	1(1.5)	16(24.6)	28(43.1)	20(30.8)	
Secondary or higher education	0(0)	20(31.3)	23(35.9)	21(32.8)	
Occupation of women					
House wife	3(0.8)	84(23.5)	125(34.9)	146(40.8)	0.978
Employed	0(0)	7(21.9)	11(34.4)	14(43.8)	
Parity					
Primiparous	0(0)	29(32.2)	34(37.8)	27(30)	**0.026**
Second screen	2(1.3)	34(22.2)	59(38.6)	58(37.9)	
Multiparous^҂^	1(0.7)	28(19)	43(29.3)	75(51)	
Mode of delivery					
Low channel	2(0.6)	81(26)	111(35.6)	118(37.8)	**0.018**
Caesarean section	1(1.3)	10(12.8)	25(32.1)	42(53.8)	
Previous abortion					
No abortion	1(0.3)	77(26.2)	106(36.1)	110(37.4)	**0.041**
1 abortion	2(2.8)	11(15.5)	20(28.2)	38(53.5)	
2 abortions	0(0)	3(14.3)	9(42.9)	9(42.9)	
3 abortions	0(0)	0(0)	1(25.0)	3(75.0)	

Note: values are expressed in terms of number and percentage. ^*∗*^Fisher's exact test. A *p* value < 0.05 is considered significant. ҂: (Babinski A, Kerenyi T, Torok O, Grazi V, Lapinski RH, Berkowitz RL. Perinatal outcome in grand and great grand multiparity: effect of parity on obstetric risk factors. Am J Obstet Gynecol 1999; 181(3):669–74). The multiparous is a woman whose parity is between 2 and 5.

## Data Availability

Access to data is restricted in order to respect the rights of third parties and the confidentiality of participants.

## References

[1] Organisation Mondiale de la Santé (2003). Obésitè: prévention et prise en charge de l‘èpidèmie mondiale. *Rapport d’une Consultation de l’OMS*.

[2] World Health Organization (2018). *Obesity and Overweight*.

[3] OMS (2020). Obésité et surpoids Pricipaux faits. https://www.who.int/fr/news-room/fact-sheets/detail/obesity-and.overweight#:~:text=Globalement%2C%20environ%2013%25%20de%20la,mondial%20entre%201975%20et%202016.

[4] Chevallier L. (2009). *Nutrition: Principes et Conseils*.

[5] Laraia B. A., Siega-Riz A. M., Dole N., London E. (2009). Pregravid weight is associated with prior dietary restraint and psychosocial factors during pregnancy. *Obesity*.

[6] Bogaerts A., Devlieger R., Van den Bergh B. R., Witters I. (2014). Obesity and pregnancy, an epidemiological and intervention study from a psychosocial perspective. *Facts, views & vision in ObGyn*.

[7] Maisonneuve E. (2011). Obésité et grossesse: revue des risques et de la prise en charge obstétricale. *Revue Médecine Périnatale*.

[8] Metwally M., Ong K., Ledger W., Li T. (2008). Does high body mass index increase the risk of miscarriage after spontaneous and assisted conception? A meta-analysis of the evidence. *Fertility and Sterility*.

[9] Sebire N., Jolly M., Harris J. (2001). Maternal obesity and pregnancy outcome: a study of 287 213 pregnancies in London. *International Journal of Obesity*.

[10] Hamon C., Fanello S., Catala L., Parot E. (2005). Conséquences de l’obésité maternelle sur le déroulement du travail et l’accouchement à l’exclusion des autres pathologies pouvant modifier la prise en charge obstétricale. *Journal de Gynecologie Obstetrique et Biologie de la Reproduction*.

[11] Cedergren M. I. (2009). Non-elective caesarean delivery due to ineffective uterine contractility or due to obstructed labour in relation to maternal body mass index. *European Journal of Obstetrics & Gynecology and Reproductive Biology*.

[12] Gunderson E. P. (2009). Childbearing and obesity in women: weight before, during, and after pregnancy. *Obstetrics & Gynecology Clinics of North America*.

[13] Gaillard R., Steegers E. A. P., Duijts L. (2014). Childhood cardiometabolic outcomes of maternal obesity during pregnancy. *Hypertension*.

[14] Dyer J. S., Rosenfeld C. R. (2011). Metabolic imprinting by prenatal, perinatal, and postnatal overnutrition: a review. *Seminars in Reproductive Medicine*.

[15] Cedergren M. I. (2004). Maternal morbid obesity and the risk of adverse pregnancy outcome. *Obstetrics & Gynecology*.

[16] Siega-Riz A. M., Viswanathan M., Moos M. K. (2009). A systematic review of outcomes of maternal weight gain according to the Institute of Medicine recommendations: birthweight, fetal growth, and postpartum weight retention. *American Journal of Obstetrics and Gynecology*.

[17] Ministère de la santé (2018). Rapport de l’enquête nationale sur les facteurs de risque communs des maladies non transmissibles, STEPS, 2017-2018. https://www.who.int/ncds/surveillance/steps/STEPS-REPORT-2017-2018-Morocco-final.pdf.

[18] Barich F., Zahrou F. E., Laamiri F. Z. (2018). Association of obesity and socioeconomic status among women of childbearing age living in urban area of Morocco. *Journal of Nutrition and Metabolism*.

[19] Wang Y. (2001). Cross-national comparison of childhood obesity: the epidemic and the relationship between obesity and socioeconomic status. *International Journal of Epidemiology*.

[20] World Health Organization (2000). *Obesity: Preventing and Managing the Global Epidemic*.

[21] International Organization for Migration (2009). *Weight Gain during Pregnancy: Reexamining the Guidelines; Committee to Reexamine IOM Pregnancy Weight Guidelines; Sponsor Briefing*.

[22] World Health Organization (1995). Physical status: the use and interpretation of anthropometry technical report.

[23] https://www.acog.org/en/clinical/clinical-guidance/committee-opinion/articles/2015/10/the-apgar-score.

[24] Ravaoarisoa L., Toy M. A. T., Raobijaona H. S., Rakotomanga J. D. M. (2014). Déterminants de la mortalité néonatale précoce dans la maternité de Befelatanana. *Antananarivo*.

[25] Ducarme G., Rodrigues A., Aissaoui F., Davitian C., Pharisien I., Uzan M. (2007). Grossesse des patientes obèses: quels risques faut-il craindre?. *Gynecologie Obstetrique & Fertilite*.

[26] Tjepkema M. (2009). Nutrition: résultats de l’enquête sur la santé dans les collectivités canadiennes. L’obésité chez les adultes au Canada: poids et grandeur mesurés. https://www.statcan.gc.ca/pub/82-620-m/2005001/article/adults-adultes/8060-fra.htm.

[27] Einarson T. R., piwko C., Koren G. (2013). Prevalence of nausea and vomiting of pregnancy in the USA: a meta analysis. *Journal of population therapeutics and clinical pharmacology = Journal de la therapeutique des populations et de la pharmacologie clinique*.

[28] Gaillard R., Durmuş B., Hofman A., Mackenbach J. P., Steegers E. A. P., Jaddoe V. W. V. (2013). Risk factors and outcomes of maternal obesity and excessive weight gain during pregnancy. *Obesity*.

[29] Mamun A. A., Mannan M., Doi S. A. R. (2014). Gestational weight gain in relation to offspring obesity over the life course: a systematic review and bias-adjusted meta-analysis. *Obesity Reviews*.

[30] Poston L., Harthoorn L. F., Van Der Beek E. M. (2011). Obesity in pregnancy: implications for the mother and lifelong health of the child, a consensus statement. *Pediatric Research*.

[31] Denison F., Price J., Graham C., Wild S., Liston W. (2008). Maternal obesity, length of gestation, risk of postdates pregnancy and spontaneous onset of labour at term. *BJOG: An International Journal of Obstetrics and Gynaecology*.

[32] Vahratian A., Zhang J., Troendle J. F., Savitz D. A., Siega-Riz A. M. (2004). Maternal prepregnancy overweight and Obesity and the pattern of labor progression in term nulliparous women. *Obstetrics & Gynecology*.

[33] Le Thai N., Lefevre G., Stella V., Vauthier D., Sfoggia D., Goulon V. (1992). Grossesse et obésité, à propos d’une étude cas témoins de 140 cas. *Journal de Gynécologie Obstétrique et Biologie de la Reproduction*.

[34] Garabedian C., Servan-Schreiber E., Rivière O., Vendittelli F., Deruelle P. (2016). Maternal obesity and pregnancy: evolution of prevalence and of place of birth. *Journal de Gynécologie Obstétrique et Biologie de la Reproduction*.

[35] Sellam R. J., Bour A. (2014). Etat nutritionnel chez des femmes de l’oriental marocain (pr2fecture d’Oujda-Angad). *American Journal of Clinical Nutrition*.

[36] Kuczmarski R. J., Guiguet M., Chau N. P., Wells J. A., Valleron A. J. (1992). Prevalence of overweight and weight gain in the United States. *International Journal of Obesity and Related Metabolic Disorders: Journal of the International Association for the Study of Obesity*.

[37] Laurier F., Wild S., Liston W. (1992). Prevalence of obesity: a comparative survey in France, the United Kingdom and the United States. *BJOG: An International Journal of Obstetrics and Gynaecology*.

[38] Garbaciak J. A., Richter M., Miller S., Barton J. J. (1985). Maternal weight and pregnancy Complications. *American Journal of Obstetrics and Gynecology*.

[39] Lashen H., Fear K., Sturdee D. W. (2004). Obesity is associated with increased risk of first trimester and recurrent miscarriage: matched case-control study. *Human Reproduction*.

[40] O’Brien T. E., Ray J. G., Chan W. S. (2003). Maternal body mass index and the risk of preeclampsia: a systematic overview. *Epidemiology*.

[41] Lashen H., Fear K., Sturdee D. W. (2004). Obesity is associated with increased risk of first trimester and recurrent miscarriage: matched case-control study. *Journal of Maternal-Fetal and Neonatal Medicine*.

[42] Rotterdam ESHRE/ASRM-Sponsored PCOS Consensus Workshop Group (2004). Consensus on diagnostic criteria and long-termhealth risks related to polycysticovary syndrome. *Fertility and Sterility*.

[43] Chen N., McNiff C., Madan J. (2010). Maternal obesity and pregnancy outcome: a study of 287 213 pregnancies in London. *International Journal of Obesity*.

[44] Duthay I., Marchetta (1991). L’accouchement des femmes obèses. *Mémoire: Ecole de Sage-Femme*.

[45] Simko M., Totka A., Vondrova D. (2019). Maternal body mass index and gestational weight gain and their association with pregnancy complications and perinatal conditions. *International Journal of Environmental Research and Public Health*.

[46] Schack-Nielsen J. L., Michaelsen F. D., Gamborg M. (2010). Obesity, obstetric complications and cesarean delivery rate-a population-based screening study. *American Journal of Obstetrics and Gynecology*.

[47] Galtier Dereure F., Boegner Lemoine C., Bringer J. (2000). Obesity and pregnancy: complications and cost. *American Journal of Clinical Nutrition*.

[48] Weiss J. L., Malone F. D., Emig D. (2004). FASTER Research Consortium Obesity obstetric complications and cesarean delivery rate—a population based screening study. *American Journal of Obstetrics and Gynecology*.

[49] de Ferranti H., Crane J., Farine D. (2002). Screening for gestational diabetes mellitus. SOGC clinical practice guideline. *Clinical Chemistry*.

[50] Bihen S. S., Choleau C., Cohen K. L. (2007). Insulinorésistance et complications métaboliques: ce que l’obésité morbide apprend au médecin. *Obstetrics & Gynecology*.

[51] Ferranti S. S., Mozaffarian D. (2008). The perfect storm: obesity, adipocyte dysfunction, and metabolic consequences. *Clinical Chemistry*.

[52] Kim P., Zhu Y., Grantz KL. (2016). Obésité et grossesse. *Gynecologie Obstetrique & Fertilite*.

[53] Huda S. S., Forrest R., Paterson N., Jordan F., Sitar N., Freeman D. J. (2014). In preeclampsia, maternal third trimester subcutaneous adipocyte lipolysis is more resistant to suppression by insulin than in healthy pregnancy. *Hypertension*.

[54] Deruelle (2011). Obésité et grossesse. *Gynecologie Obstetrique & Fertilite*.

[55] Hamdaoui M., Sakly R., Alguemi C. C., Bennour A., Jallouli K., Doghri T. (1990). Anémie nutritionnelle de la femme enceinte dans la région de Kairouan (Tunisie). *Colloque INSERM*.

[56] Baidy B. L. O., Kone Y., Bassirou L. Y. (1996). Anémie nutritionnelle de la grossesse à Nouakchott. *Medecine d’Afrique Noire*.

[57] mondiale B. (2017). Un cadre d’investissement pour l’atteinte des cibles mondiales de nutrition: l’anémie. https://openknowledge.worldbank.org/bitstream/handle/10986/26069/Anemia_french_WEB.PDF?sequence=25.

[58] OMS et UNICEF (2018). Cadre mondial de suivi de la nutrition guide pratique pour le suivi des avancées par rapport aux cibles mondiales 2025. https://file:///C:/Users/admin/Downloads/9789242513608-fre.pdf.

